# Transcriptomic and Proteomic Approaches Reveal the Biological Functions of Two Novel Porcine-Origin Noncoding DNA Molecules

**DOI:** 10.1155/2023/5909892

**Published:** 2023-11-23

**Authors:** Libin Wen, Jianping Xie, Qi Xiao, Kongwang He

**Affiliations:** ^1^Institute of Veterinary Medicine, Jiangsu Academy of Agricultural Sciences, Nanjing 210014, China; ^2^Key Laboratory of Veterinary Biological Engineering and Technology, Ministry of Agriculture, Nanjing 210014, China; ^3^Jiangsu Co-innovation Center for Prevention and Control of Important Animal Infections Diseases and Zoonoses, Yangzhou University, Yangzhou 225009, China; ^4^Jiangsu Key Laboratory for Food Quality and Safety—State Key Laboratory Cultivation Base of Ministry of Science and Technology, Nanjing 210014, China; ^5^GuoTai (Taizhou) Center of Technology Innovation for Veterinary Biologicals, Taizhou 225300, China

## Abstract

Porcine circovirus-like mini agents (PCVLs) with small circular noncoding DNA genomes have recently been discovered in animals. Currently, the biological activity of PCVLs remains unclear. In this study, we conducted transcriptomic and proteomic analyses to compare the differential expression of genes and proteins in the livers of PCVL258/PCVL264 molecular clone-infected and mock-infected BALB/c mice at 7 and 28 days postinfection (dpi). Gene Ontology/Kyoto Encyclopedia of Genes and Genomes association analysis of the transcriptome and proteome showed that the differentially expressed genes and proteins (DEGs and DEPs) in the livers of PCVL258-infected mice were predominantly enriched in metabolic, cancer, and neurodegenerative disease signaling pathways. On the other hand, the DEGs and DEPs in the livers of PCVL264-infected mice were principally related to metabolic, reproductive, and pancreatic-associated pathways. We present the first application of transcriptomics combined with proteomics to determine the biological activities of small pathogen-associated DNA molecules, thus providing valuable information for understanding small circular DNA molecules that cannot encode proteins in the generation of specific human diseases.

## 1. Introduction

Porcine circoviruses (PCVs), belonging to the genus Circovirus of the family *Circoviridae*, are small nonenveloped viruses (17–22 nm in diameter) with single-stranded circular DNA (∼2,000 nt). To date, four types of PCVs (PCV1–PCV4) have been identified [1–5]. PCVs have been associated with multiple disease syndromes in pigs, including postweaning multisystemic wasting syndrome (PMWS) and reproductive disorders. Pigs with PMWS exhibit poor growth rates, reduced thriving, and wasting. Porcine circovirus-associated diseases have traditionally been referred to as those associated with PCV2 infection [6, 7]. PCV2 infection has been reported in nearly every country and has caused significant economic losses to the swine industry worldwide.

In 2019, four PCV-like mini agents were reported in China. Their nucleotide lengths were 258, 264, 201, and 347 nt. Two agents (PCVL258 and PCVL264) were from PMWS-affected pigs, and the other two (PCVL201 and PCVL347) were from clinically healthy cattle [8]. Subsequently, PCVL258 and PCVL264 were detected in the sera of dogs with respiratory or digestive tract symptoms, and PCVL258 was confirmed in the sera of cats [9]. The four PCV-like mini agents had a small circular genome that did not encode any protein; they showed 81.3%–98.5% nucleotide sequence identity over the entire genome and 43.3%–95.5% identity with PCV1, PCV2, and PCV-like viruses [10, 11]. These agents appear to be products of a natural recombination event between PCV2 (or PCV-like viruses) and other molecules. Interestingly, porcine circovirus-like mini agents (PCVLs) can replicate independently without needing PCV as a helper virus, and both types of viruses can infect animals on their own.

In recent years, proteomics and transcriptomics have become effective tools for evaluating and predicting the activities of newly discovered biological factors using big data analysis. Integration of transcriptomic and proteomic approaches can provide more accurate information. To date, little is known regarding the effects of these DNAs on the host. In the present study, two porcine-origin DNAs (PCVL258 and PCVL264) were selected and synthesized by genomic molecular cloning with double copies in tandem and then inoculated into BALB/c mice. Alterations in the transcriptome and proteome in the liver were determined to compare the differences in gene or protein expression levels after infection with the two DNAs. Bioinformatic analysis indicated that these differentially expressed genes (DEGs) or proteins (DEPs) were involved in various signaling pathways, and a protein–protein network was constructed based on the two DNAs. For the first time, this study analyzes transcriptome and proteome changes in animals after infection with two porcine-origin DNAs (PCVL258 and PCVL264). Our findings provide valuable information to determine the potential functions of PCVL258 and PCVL264.

## 2. Materials and Methods

### 2.1. Construction of PCVL258 and PCVL264 Infectious Molecular Clones

**Figure 1:**
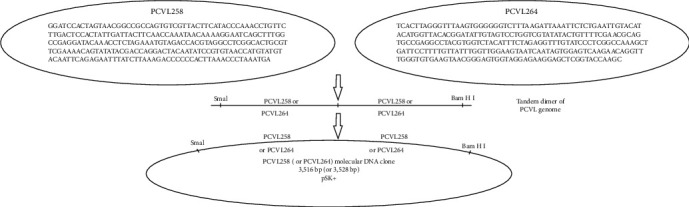
Construction of infectious PCVL258 or PCVL264 molecular DNA clones. Tandem genome dimers of PCVL258 or PCVL264 were synthesized according to the published sequence of PCVL258 (KY270811) or PCVL264 (KY270812). Full-length PCVL258 and PCVL264 genomes are shown in the figure. The molecular DNA clones with Sma I and BamH I restriction enzyme sites at the 5ʹ and 3ʹ ends were cloned into the pBluescript II SK (pSK) plasmid to produce molecular PCVL258 and PCVL264 clones, respectively.

The tandem genome dimers of full-length PCVL258 and PCVL264 were synthesized by Genscript (Nanjing, China) according to the published sequences (KY270811 and KY270812) derived from the sera of piglets with a PMWS-like disease and were then cloned into the pBluescript SK (pSK) vector (Stratagene, La Jolla, CA) (Figure 1). The recombinant plasmids were sequenced to confirm that no incorrect nucleotides or new open-reading frames (ORFs) were introduced.

### 2.2. Experimental Design

Forty-five BALB/c mice 4 weeks of age were randomly assigned to three groups of 15 animals each. Before inoculation, the mice were confirmed to be negative for PCV1, PCV2, PCVL258, and PCVL264 using polymerase chain reaction (PCR). Mice in group 1 were administered 100 *µ*g of empty vector pSK by intramuscular injection and were used as the control group (C). Mice in group 2 were inoculated intramuscularly with recombinant plasmid DNA of the PCVL258 molecular clone (TII), and mice in group 3 were inoculated with pSK-PCVL264 (T). Each mouse was injected with 100 *µ*g of recombinant plasmid DNA. The mice were monitored daily for clinical signs of the disease (including temperature, body weight, diet, and mental state). Three mice were randomly selected from each group and euthanized by intraperitoneal injection of a lethal dose of pentobarbital at 3, 7, 14, 21, and 28 days postinoculation (dpi). Various tissues and organs (heart, liver, spleen, lung, and kidney) were collected for PCR analysis. Livers collected at 7 (nos. 1, 2, and 3) and 28 (nos. 4, 5, and 6) dpi were used for omics analysis.

### 2.3. Ethics Statement

All experimental protocols were approved by the Committee on the Ethics of Animal Experiments at the Institute of Veterinary Medicine, Jiangsu Academy of Agricultural Sciences (JAAS No 20100604). All methods followed the guidelines and regulations of Jiangsu Province Animal Regulations (Government Decree No. 45). Further, all methods followed the ARRIVE guidelines (https://arriveguidelines.org) for reporting animal experiments.

### 2.4. DNA Extraction and PCR

According to the manufacturer's instructions, DNA from mouse tissue samples (heart, liver, spleen, lung, and kidney) was extracted using a commercial DNA extraction kit (Tiandz, Inc., China). PCR for PCVL258 and PCVL264 was performed as previously described [8]. Meanwhile, the extracted DNA was subjected to PCR using T7 (5ʹ-AATACGACTCACTATAG-3ʹ) and T3 (5ʹ-ATTAACCCTCACTAAAG-3ʹ) primers. The amplified products were electrophoresed on a 2% agarose gel and visualized by staining with 0.5 *µ*g/mL of ethidium bromide. The PCR products were purified using a gel extraction Kit (AXYGEN, China) according to the manufacturer's instructions, cloned into the pMD18T vector (TaKaRa, Dalian, China), and sequenced for verification.

### 2.5. Transcriptome Sequencing and Analysis

Total RNA was extracted from liver tissue using a Trizol reagent kit (Invitrogen, Carlsbad, CA, USA). RNA quality was assessed using an Agilent 2100 Bioanalyzer (Agilent Technologies, Palo Alto, CA, USA) and checked using RNase-free agarose gel electrophoresis. The mRNA was enriched using Oligo (dT) beads (Illumina), broken into short fragments, and then reverse transcribed into first-strand cDNA with random hexamers further used to synthesize second-strand cDNA. A cDNA library was constructed through cDNA purification, end-repair, poly(A) addition, and ligation to Illumina sequencing adapters. The library was sequenced using the Illumina HiSeq^TM^ 2500 platform (Gene Denovo Biotechnology Co., Guangzhou, China).

To obtain high-quality clean reads, the sequencing raw reads were filtered by fastp (version 0.18.0) to remove reads containing adapters, unknown nucleotides (>10%), low-quality (*q*-value ≤20) bases (>50%), all A-bases, and ribosomal RNA. The clean reads were mapped to the reference genome using HISAT2.2.4.

### 2.6. Proteome Sequencing and Analysis

Liver tissues were ground into powder in liquid nitrogen, transferred into lysis buffer containing 1% SDS, 8 M urea, and 1 mg/mL protease inhibitor cocktail, vortexed, and lysed for 30 min on ice. The samples were then homogenized and centrifuged to collect the supernatant. The protein concentrations of the samples were measured using a bicinchoninic acid assay (BCA) protein quantitation kit (Thermo, USA). The protein sample was mixed with Tris(2-carboxyethyl) phosphine (TCEP) (0.5 M) and iodoacetamide (1 M) to cleave disulfide linkages and reductive alkylation. Then, the protein sample was digested by sequence-grade modified trypsin (Promega, Madison, WI), and the digested peptide mixture was desalted using C18 ZipTip, quantified by the Pierce™ Quantitative Colorimetric Peptide Assay, and lyophilized using a SpeedVac. The resultant peptide mixture was labeled using an iTRAQ-8Plex Isobaric Mass Tag Labeling Kit (Thermo Fisher Scientific, MA, USA) following the manufacturer's instructions. The labeled peptide mixture was subjected to high-pH reverse phase separation, and the separated peptide fractions were analyzed by nano-HPLC-MS/MS in data-dependent acquisition mode, with automatic switching between MS and MS/MS modes. Raw data-independent acquisition (DIA) data with default parameters were processed and analyzed using Spectronaut 15 (Biognosys AG, Switzerland).

### 2.7. Identification of DEGs/DEPs and Data Analysis

RNA differential expression analysis was performed using DESeq2 software to compare two groups (and edgeR between two samples). Genes/transcripts with a false discovery rate (FDR) below 0.05 and a threshold |log2 fold change (FC)| ≥ 1 were considered differentially expressed genes/transcripts (DEGs).

Protein differential expression analysis was performed by Mascot (the protein sequencing method used ITRAQ/TMT)/MaxQuant (the protein sequencing method uses Label-free)/Spectronaut Pulsar (the protein sequencing method uses DIA) on two different groups or samples. The protein quality control criterion was a predictor threshold of 1.0% FDR. A decoy database was generated using a mutated strategy, similar to scrambling amino acid sequences with a random number (at least two amino acids and up to half the total length of peptides). Spectronaut was used for automatic correction, and all MS1 peptides that met the screening conditions were used to calculate expression levels. The average peak areas of the first three MS1 peptides with an FDR of less than 1.0% were screened for protein quantification.

Student's *t*-test was used for protein abundance analysis. Normalization was performed by averaging the abundances of all peptides. Medians were used for averaging. For accurate comparison between samples, protein quantification data with an FC ≥1.5 and *P* < 0.05 (where the protein sequencing method used DIA) were identified as differentially expressed proteins (DEPs).

### 2.8. Gene Ontology (GO) Term and Kyoto Encyclopedia of Genes and Genomes (KEGG) Pathway Enrichment Analysis

DEGs and DEPs were analyzed using the GO database (http://www.geneontology.org), which features the main categories of biological processes (BPs), molecular functions (MFs), and cellular components (CCs). The KEGG online service tool (http://www.genome.jp/kaas-bin/kaas_main) was used to annotate the submitted genes/proteins, and the annotated genes/proteins were then matched to the corresponding pathways in the databases using the KEGG mapper (http://www.kegg.jp/kegg/mapper.html). The *P*-value calculated using a hypergeometric test was subjected to FDR correction with FDR ≤ 0.05. GO terms and pathways meeting this criterion were defined as significantly enriched.

### 2.9. GO/KEGG Association Analysis of Transcriptome and Proteome

The correlation analysis between GO functions and KEGG pathway information in the transcriptome and proteome was performed in R (version 3.5.1). A nine-quadrant map was drawn based on changes in the expression of genes in the transcriptome and proteome. The mRNA levels in the third and seventh quadrants were consistent with the corresponding DEPs, indicating that a gene was not regulated or less regulated at the posttranscriptional and translational levels.

### 2.10. Protein–Protein Interaction (PPI) Network Construction

A PPI network was identified using the STRING database (http://www.string-db.org/), where genes were considered nodes and interactions as edges in a network. The network file was visualized using the Cytoscape software to identity core and hub gene interactions. The interactions with a combined score over 0.4 were considered statistically significant. In the generated networks, nodes represented proteins, and edges represented their interactions.

### 2.11. Real-Time RT-PCR Validation of Differentially Expressed Genes

cDNA was synthesized using reverse transcriptase (Promega), oligo(dT), and random primers with 5 *μ*g RNA treated with DNase I (Ambion) from the same liver samples at 28 dpi used for PCVL258 biological activity research as those used in the transcriptomic analysis and proteomic analysis. Eight genes (four with upregulated expressions and four with downregulated expressions) involved in the human disease pathway were randomly selected. These genes were *Try4* (trypsin 4), *H4c17* (H4 clustered histone 17), *Prss2* (protease serine 2), *H2bc3* (H2B clustered histone 3), *Cyp2b10* (cytochrome P450 family 2 subfamily b polypeptide 10), *Gsta1* (glutathione S-transferase alpha 1), *Hba-a2* (hemoglobin alpha, adult chain 2), and *Cyp17a1*(cytochrome P450 family 17 subfamily A member 1). GAPDH was used as an endogenous control. Quantitative RT-PCR (qRT-PCR) was performed using the SYBR Green I dye. PCRs were performed using gene-specific primers (Table 1). The cycling conditions consisted of an initial denaturation step of 5 min at 95°C, followed by 40 cycles of 15 s at 95°C and 30 s at 60°C. qPCR amplifications were performed in triplicate for each sample. Melting curves were analyzed to ensure that a single amplicon was produced for the target gene. FC values were calculated using the 2^−*ΔΔ*Ct^ method.

## 3. Results

### 3.1. Tissue Distribution of PCVL258 and PCVL264

None of the mice had an obvious clinical syndrome during the experimental period, and no apparent gross lesions were observed in any of the mice at necropsy.

PCVL258 and PCVL264 molecular DNA clones were infectious when injected directly into the muscles of 4-week-old BALB/c mice. The results showed that even if the pSK vector sequence could not be amplified by PCR because of its degradation in vivo, the nucleotide fragments of PCVL258 or PCVL264 could still be amplified. Tissue samples were collected from all control and inoculated mice at 3, 7, 14, 21, and 28 dpi. All tissues from control mice were negative for PCVL258 and PCVL264 throughout the study. PCVL258 DNA was detected in group TII mice's heart, liver, spleen, lungs, and kidneys on each dpi. Similarly, PCVL264 was detected to varying degrees in group T mice's heart, liver, spleen, lungs, and kidneys on each dpi (Table 2). The sequences of the PCR products amplified from the selected tissues were identical to those of the molecular DNA clones.

### 3.2. Analysis of DEGs and DEPs for PCVL258 and PCVL264 at 7 dpi

According to alignment against the reference sequences, 16,682 genes were identified, and the transcription of 2,154 genes in the livers of PCVL258-infected mice at 7 dpi was altered dramatically, comprising 722 genes with upregulated expressions and 1,432 genes with downregulated expressions. In total, 4,815 proteins were successfully detected, and 1,526 proteins were quantified. Of these proteins, the expressions of 828 were significantly upregulated, and 698 were significantly downregulated in liver cells at 7 dpi. Integration analysis of common genes between the transcriptome and proteome showed 2,928 genes and 4,785 proteins.

GO enrichment analysis was performed to evaluate the biological functions after infection with PCVL258. In the BP category, DEGs were enriched in cellular process, metabolic process, biological regulation, regulation of biological process, and response to stimulus, and the DEPs were enriched in cellular process, single-organism process, metabolic process, biological regulation, and regulation of biological process. Cell, cell part, and organelle contained the most DEGs/DEPs in the CC category. In the MF category, binding and catalytic activity were the most abundant functions of DEGs/DEPs.

The DEGs were enriched in KEGG pathways involved in metabolic pathways, pancreatic secretion, steroid hormone biosynthesis, herpes simplex infection, chemical carcinogenesis, retinol metabolism, glycerolipid metabolism, protein digestion and absorption, circadian rhythm, PPAR signaling pathway, metabolism of xenobiotics by cytochrome P450, drug metabolism-other enzymes, linoleic acid metabolism, cholesterol metabolism, nicotinate and nicotinamide metabolism, peroxisome, amino sugar, and nucleotide sugar metabolism, fat digestion and absorption, and hepatocellular carcinoma. The DEPs were enriched in metabolic pathways, metabolism (carbon, alanine, aspartate, glutamate, tryptophan, pyruvate, D-glutamine and D-glutamate, cysteine and methionine, glycine, serine, and threonine, seleno-compound, 2-oxo carboxylic acid, propanoate, tyrosine, ascorbate and aldarate, starch and sucrose, and glutathione), arginine biosynthesis, ribosomes, sulfur relay systems, chemical carcinogenesis, and Parkinson's disease.

A total of 22,372 genes were identified, and the transcription levels of 10 genes in the livers of PCVL264-infected mice at 7 dpi were dramatically altered, and their expression was downregulated. In total, 6,430 proteins were successfully detected, and 10 were quantified. The expressions of three proteins were significantly upregulated, and those of seven were downregulated at 7 dpi.

GO enrichment analysis was performed to evaluate the biological functions after infection with PCVL264. DEGs were enriched in single-organism process, cellular, and metabolic process in the BP category. Cell, cell part, and organelle contained the most DEGs in the CC category. In the MF category, binding and nucleic acid-binding transcription factor activity were the most enriched functions. The DEPs were enriched in metabolic process, biological regulation, cellular process, developmental process, immune system process, localization, and single-organism process in the BP category; in the extracellular region, extracellular region part, cell, and cell part in the CC category; and binding and catalytic activity in the MF category.

The five downregulated DEGs were enriched in KEGG pathways involved in the Jak-STAT signaling pathway, the prolactin signaling pathway, the cell cycle, signaling pathways regulating the pluripotency of stem cells, and the mitogen-activated protein kinase (MAPK) signaling pathway. KEGG pathway analysis revealed that these DEPs were enriched in carbohydrate digestion and absorption, pancreatic secretion, starch, sucrose metabolism, malaria, protein digestion and absorption, nitrogen metabolism, collecting duct acid, gastric acid, and bile secretion.

### 3.3. Analysis of DEGs and DEPs for PCVL258 and PCVL264 at 28 dpi

In total, 16,544 genes were identified, and the transcription of 974 genes in the livers of PCVL258-infected mice at 28 dpi were identified as DEGs, comprising 307 genes with upregulated expressions and 667 genes with downregulated expressions. A total of 5,946 proteins were successfully detected, and there were significant changes in 620 proteins, of which the expressions 273 were upregulated and 347 downregulated at 28 dpi. Integration analysis of common genes between transcriptome and proteome showed 3,569 genes and 5,915 proteins.

The BP, CC, and MF aspects of the DEGs/DEPs were analyzed using the GO database. For BP annotation, DEGs in the livers of PCVL258-infected mice at 28 dpi were involved in cellular process, biological regulation, metabolic process, regulation of biological process, and response to stimulus; the DEPs were involved in cellular process, single-organism process, metabolic process, and biological regulation. For CC annotation, the DEGs/DEPs were localized to the cell, cell part, organelle, membrane, and organelle part. Moreover, the DEGs/DEPs in the MF category were primarily involved in binding and catalytic activity.

The DEGs that were enriched in KEGG pathways involved metabolism (metabolic pathways, metabolism of xenobiotics by cytochrome P450, glutathione metabolism, drug metabolism-other enzymes, drug metabolism-cytochrome P450, biosynthesis of unsaturated fatty acids, primary bile acid biosynthesis, other glycan degradation, and retinol metabolism), organismal systems (bile secretion, circadian rhythm, and PPAR signaling pathway), and human diseases (hepatocellular carcinoma, insulin resistance, and pathways in cancer). The DEPs that were enriched in KEGG pathways were mainly involved in arginine biosynthesis, alanine, aspartate, and glutamate metabolism, systemic lupus erythematosus, D-glutamine and D-glutamate metabolism, metabolic pathways, ribosomes, biosynthesis of amino acids, transcriptional misregulation in cancers, pyrimidine metabolism, and carbon metabolism.

In total, 22,372 genes were identified, and the transcription levels of 584 genes in the livers of PCVL264-infected mice at 28 dpi were dramatically altered, with 309 genes with upregulated expressions and 275 genes with downregulated expressions. A total of 6,430 proteins were successfully detected, and there were significant changes in 38 proteins, of which the expressions of 14 were upregulated, and those of 24 were downregulated at 28 dpi.

The BPs, CCs, and MFs of the DEGs/DEPs were analyzed using the GO database. For BP annotation, the DEGs in the livers of PCVL264-infected mice at 28 dpi were involved in cellular, metabolic, response to stimulus, and single-organism processes. The DEGs were localized to the cell, cell part, and organelle for CC annotation. The DEGs in the MF category involved binding and catalytic activity. The DEPs were enriched in the carbohydrate metabolic process, lipid metabolic process, cell–cell signaling, mitochondrion organization, homeostatic process, regulation of biological quality, generation of precursor metabolites and energy, and response to stimulus in the BP category; in extracellular space, extracellular region part, extracellular region, and endoplasmic reticulum in the CC category; and catalytic activity, hydrolase activity, acting on glycosyl bonds, kinase activity, oxidoreductase activity, transferase activity, and transferring phosphorus-containing groups in the MF category.

The 206 DEGs were enriched in KEGG pathways involved in the cell cycle, progesterone-mediated oocyte maturation, the FoxO signaling pathway, homologous recombination, the p53 signaling pathway, ribosome biogenesis in eukaryotes, longevity regulating pathway, insulin resistance, oocyte meiosis, the Fanconi anemia pathway, protein processing in the endoplasmic reticulum, the estrogen signaling pathway, and antigen processing and presentation. KEGG pathway analysis showed that the DEPs were enriched in starch and sucrose metabolism, carbohydrate digestion and absorption, biosynthesis of unsaturated fatty acids, complement and coagulation cascades, metabolic pathways, steroid hormone biosynthesis, fatty acid metabolism, nicotinate and nicotinamide metabolism, biosynthesis of secondary metabolites, arachidonic acid metabolism, phenylalanine, tyrosine, and tryptophan biosynthesis, and pancreatic secretion.

### 3.4. GO/KEGG Association Analysis between the Transcriptome and Proteome of PCVL258 and PCVL264

To effectively identify the key genes or proteins in the relevant pathways, correlation analysis between GO functions and KEGG pathway information in the transcriptome and proteome was performed, and the similarities and differences between gene functions and metabolic pathways in the two groups were compared.

For the GO association analysis, the DEGs and DEPs in the livers of PCVL258-infected mice at 7 dpi and in the third quadrant were enriched in single-organism process, cellular process, and metabolic process in the BP category; in cell, cell part, and organelle in the CC category; and in binding and catalytic activity in the MF category. The DEGs and DEPs in the seventh quadrant were mostly enriched in similar BP, CC, and MF categories as those in the third quadrant.

DEGs and DEPs in the livers of PCVL258-infected mice at 28 dpi and in the third quadrant were enriched in biological regulation, cellular process, metabolic process, regulation of biological process, and single-organism process within the BP category; in the extracellular region, cell, cell part, organelle, and extracellular region part within the CC category; and in binding and catalytic activity within the MF category. The DEGs and DEPs in the seventh quadrant were enriched in single-organism process, metabolic process, and cellular process within the BP category; in the cell, cell part, organelle, and organelle part within the CC category; and in binding and catalytic activity within the MF category.

**Figure 2:**
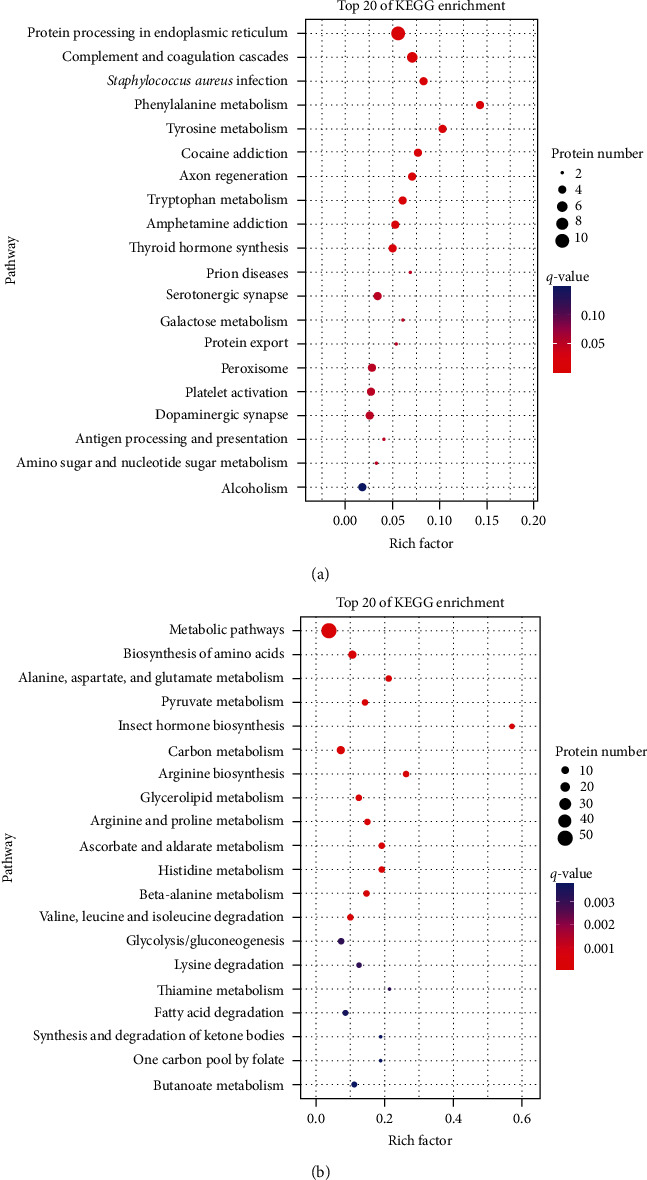
KEGG enrichment analysis for both DEGs and the corresponding DEP expression levels that were upregulated (a) or downregulated (b) in the livers of mice in the control group (C) and PCVL258-infected group (TII) at 7 days after infection. Top 20 enriched KEGG pathways.

For the association analysis, 30 DEGs and DEPs in the third quadrant of PCVL258-infected mice at 7 dpi were enriched in KEGG pathways involved in protein processing in endoplasmic reticulum, complement and coagulation cascades, *Staphylococcus aureus* infection, phenylalanine metabolism, tyrosine metabolism, cocaine addiction, axon regeneration, tryptophan metabolism, amphetamine addiction, thyroid hormone synthesis, and prion diseases. The 61 DEGs and DEPs in the seventh quadrant were mainly enriched in metabolic pathways; biosynthesis of amino acids; alanine, aspartate, and glutamate metabolism; pyruvate metabolism; carbon metabolism; arginine biosynthesis; glycerolipid metabolism; arginine and proline metabolism; ascorbate and aldarate metabolism; histidine metabolism; beta-alanine metabolism; and valine, leucine and isoleucine degradation (Figure 2).

**Figure 3:**
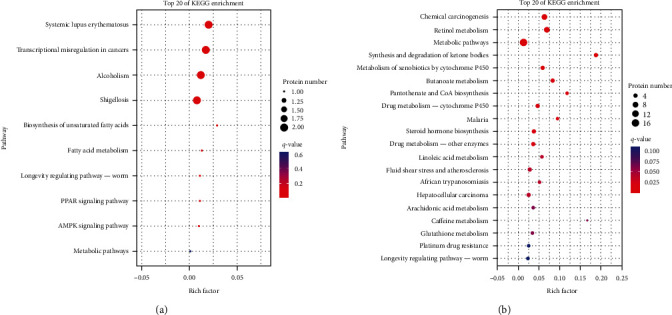
KEGG enrichment analysis for both DEGs and the corresponding DEP expression levels that were upregulated (a) or downregulated (b) in the livers of mice in the control group (C) and PCVL258-infected group (TII) at 28 days after infection. Top 20 enriched KEGG pathways are shown.

The three DEGs and DEPs in the third quadrant for PCVL258-infected mice at 28 dpi were enriched in KEGG pathways involved in systemic lupus erythematosus, transcriptional misregulation in cancers, alcoholism, shigellosis, and the biosynthesis of unsaturated fatty acids. The 22 DEGs and DEPs in the seventh quadrant were enriched in chemical carcinogenesis, retinol metabolism, metabolic pathways, synthesis and degradation of ketone bodies, metabolism of xenobiotics by cytochrome P450, butanoate metabolism, pantothenate and CoA biosynthesis, drug metabolism-cytochrome P450, steroid hormone biosynthesis, drug metabolism-other enzymes, linoleic acid metabolism, fluid shear stress and atherosclerosis, and hepatocellular carcinoma (Figure 3).

For the Go association analysis, the DEGs and DEPs in the livers of PCVL264-infected mice at 7 dpi and that in the third quadrant were enriched in cellular process, metabolic process, and single-organism process in the BP category; in cell, cell part, membrane, and organelle in the CC category; and in binding and transporter activity in the MF category. The DEGs and DEPs in the seventh quadrant were enriched in cellular process, metabolic process, and single-organism process in the BP category; in the cell, cell part, extracellular region, extracellular region part, macromolecular complex, and organelle in the CC category; and in binding and catalytic activity within the MF category.

The DEGs and DEPs in the third quadrant of PCVL264-infected mice at 28 dpi were enriched in cellular process, metabolic process, and single-organism process within the BP category, in the extracellular region and extracellular region part within the CC category, and in catalytic activity and binding within the MF category. The DEGs and DEPs in the seventh quadrant were enriched in cellular process, single-organism process, biological regulation, metabolic process, and response stimulus in the BP category, in cell, cell part, and organelle in the CC category, and in binding and catalytic activity in the MF category.

**Figure 4:**
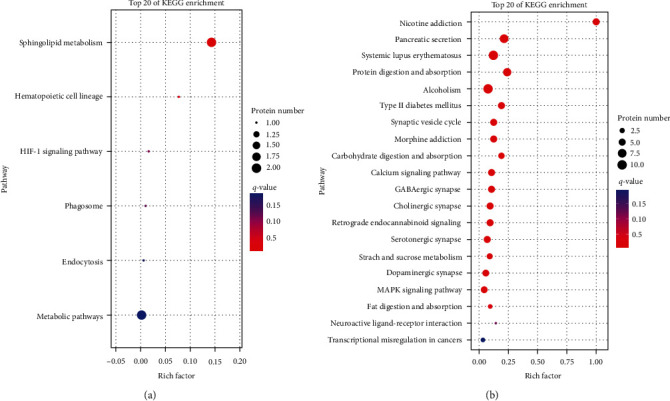
KEGG enrichment analysis for both DEGs and the corresponding DEP expression levels that were upregulated (a) or downregulated (b) in the livers of mice in the control group (C) and PCVL264-infected group (T) at 7 days after infection. Top 20 enriched KEGG pathways are shown. The *x*-axis shows the rich factor. The *y*-axis shows the pathway names. The circle area indicates the number of enriched DEGs in the pathway.

In the KO association analysis, the three DEGs and DEPs in the third quadrant for PCVL264-infected mice at 7 dpi were enriched in KEGG pathways involved in sphingolipid metabolism, hematopoietic cell lineage, HIF-1 signaling pathway, phagosome, endocytosis, and metabolic pathways. The 32 DEGs and DEPs in the seventh quadrant were enriched in nicotine addiction, pancreatic secretion, systemic lupus erythematosus, protein digestion and absorption, Type II diabetes mellitus, synaptic vesicle cycle, morphine addiction, carbohydrate digestion and absorption, calcium signaling pathway, GABAergic synapse, cholinergic synapse, retrograde endocannabinoid signaling, serotonergic synapse, starch and sucrose metabolism, dopaminergic synapse, the MAPK signaling pathway, and fat digestion and absorption (Figure 4).

**Figure 5:**
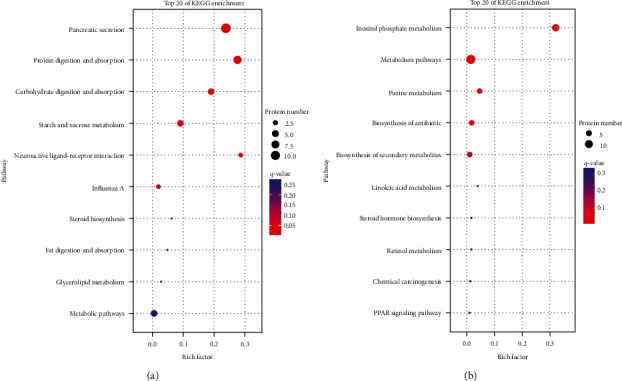
KEGG enrichment analysis for both DEGs and the corresponding DEP expression levels that were upregulated (a) or downregulated (b) in the livers of mice in the control group (C) and PCVL264-infected group (T) at 28 days after infection. The top 20 enriched KEGG pathways are shown. The *x*-axis shows the rich factor. The *y*-axis shows the pathway names. The size of each point represents the number of genes enriched in a particular pathway. The greater the value of the rich factor and the smaller the *Q*-value, the more significant the degree of enrichment.

The 13 DEGs and DEPs in the livers of PCVL264-infected mice at 28 dpi and those in the third quadrant were enriched in KEGG pathways involved in pancreatic secretion, protein digestion and absorption, carbohydrate digestion and absorption, starch and sucrose metabolism, neuroactive ligand–receptor interaction, influenza A, steroid biosynthesis, fat digestion and absorption, and glycerolipid metabolism. The 15 DEGs and DEPs in the seventh quadrant were enriched in inositol phosphate metabolism, metabolic pathways, purine metabolism, biosynthesis of antibiotics, biosynthesis of secondary metabolites, linoleic acid metabolism, steroid hormone biosynthesis, retinol metabolism, and chemical carcinogenesis (Figure 5).

### 3.5. Cluster and Interaction Network Analysis of DEPs in the Livers of PCVL258 and PCVL264-Infected Mice

To better understand the impact of PCVL258 infection, according to the transcriptomic and proteomic association analysis results for PCVL258 at 7 and 28 dpi, we selected pathways related to metabolism, neurodegenerative disease, and cancer to study the interaction network of DEPs after PCVL258 infection. The DEPs were searched using the STRING database to provide a visual network of functional relationships between DEPs using the Cytoscape software.

**Figure 6:**
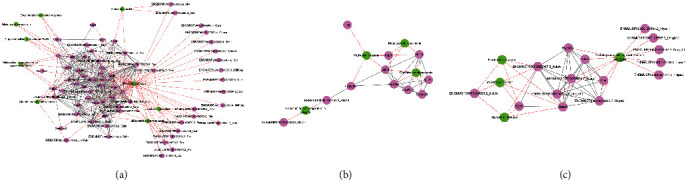
The most significant protein–protein interaction (PPI) model for the livers of mice was based on DEPs from PCVL258 infection. A green circle represents a pathway, and a pink circle represents DEP. The solid lines indicate the string relationship between proteins, and the dotted lines indicate the relationship between pathways and proteins. (a) Network of DEPs related to metabolism. (b) Network of DEPs related to cancers. (c) Network of DEPs related to neurodegenerative diseases.

The PPI network related to metabolism contained 67 nodes and 364 edges, and DEPs were enriched in metabolic pathways, carbon metabolism, purine metabolism, glutathione metabolism, steroid hormone biosynthesis, biosynthesis of amino acids, drug metabolism-other enzymes, drug metabolism-cytochrome P450, and metabolism of xenobiotics by cytochrome P450. Cancer-related PPI had 11 nodes and 33 edges, and these DEPs were enriched in pathways of cancer, chemical carcinogenesis, hepatocellular carcinoma, and transcriptional misregulation in cancers. The neurodegenerative disease-related PPI contained 15 nodes and 50 edges, and DEPs were enriched with Alzheimer's disease, Parkinson's disease, Huntington's disease, and protein processing in the endoplasmic reticulum (Figure 6).

We selected pathways related to metabolism, reproductive disorders, and insulin to study the interaction network of DEPs after PCVL264 infection based on the transcriptomic and proteomic association analysis results for PCVL264 at 7 and 28 dpi.

**Figure 7:**
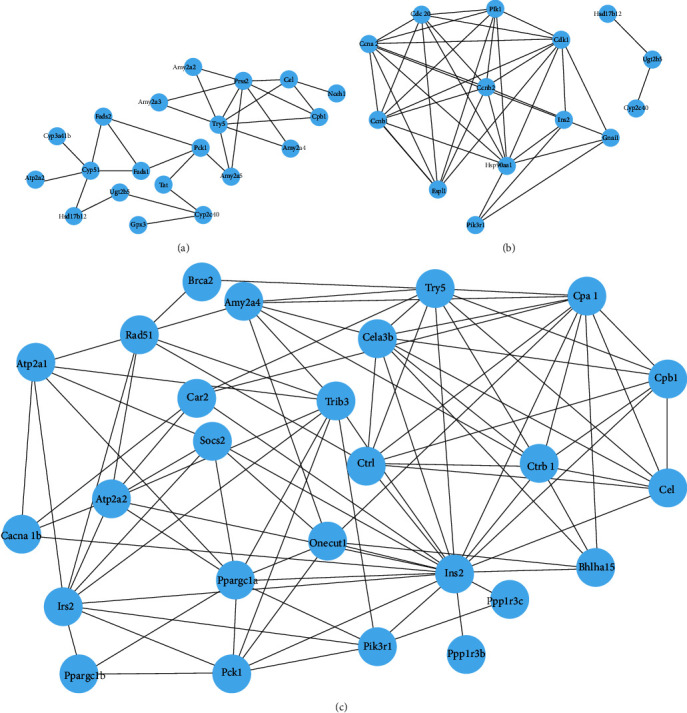
STRING software was used to construct an interaction diagram of the differentially expressed protein network to show the functional connections between differentially expressed proteins in the livers of mice after PCVL264 infection. The network nodes represent the DEPs. (a) Network of DEPs related to metabolism. (b) Network of DEPs related to reproduction. (c) Network of DEPs related to pancreatic function.

The PPI network related to metabolism and organismal systems contained 20 nodes and 29 edges, and DEPs were enriched in starch and sucrose metabolism, carbohydrate digestion and absorption, biosynthesis of unsaturated fatty acids, fatty acid metabolism, nicotinate and nicotinamide metabolism, biosynthesis of secondary metabolites, arachidonic acid metabolism, phenylalanine, tyrosine and tryptophan biosynthesis, pancreatic secretion, retinol metabolism, steroid biosynthesis, protein digestion and absorption, bile secretion, gastric acid secretion, and collecting duct acid secretion. The reproduction-related PPI network had 11 nodes and 35 edges, and these DEPs were enriched in steroid hormone biosynthesis, progesterone-mediated oocyte maturation, and oocyte meiosis. Human diseases and organismal system-related PPI comprised 26 nodes and 92 edges, and DEPs were enriched in pancreatic secretion, maturity-onset diabetes of the young, type I diabetes mellitus, type II diabetes mellitus, insulin signaling pathway, insulin resistance, and pancreatic cancer (Figure 7).

### 3.6. Validation of DEGs by qRT-PCR Analysis

To validate the expression levels of the DEGs obtained from transcriptomic and proteomic analysis, we analyzed the selected eight genes (*Try4*, *H4c17*, *Prss2*, *H2bc3*, *Cyp2b10*, *Gsta1*, *Hba-a2*, and *Cyp17a1*) using qRT-PCR. Although FC values were not equal between the qRT-PCR and transcriptome sequencing results, the gene expression patterns were concordant with the FC direction, indicating that the RNA-seq data are reliable (Table 3).

## 4. Discussion

Since the first report of PCVLs, the nature of these DNA molecules has remained unclear. Based on the current classification status of viruses, they may be classified as satellite nucleic acids or viroids. Satellite nucleic acids are molecules that encode either nonstructural proteins or no proteins at all, and they are encapsidated by the CP of helper viruses. Viroids are highly structured, circular, noncoding, single-stranded RNA molecules that induce symptoms in susceptible host plants. Viroids have not yet been found in animals, and whether the four PCV-like mini agents in animals belong to satellite DNAs or represent viroids in the form of DNA is unclear.

Cress et al. [12] demonstrated for the first time that plasmid DNAs containing potato spindle tuber viroid cDNA dimers are infectious. Similarly, recombinant DNAs containing full-length dimeric PCV2 and porcine circovirus-like virus P1 DNA were infectious [13, 14]. Currently, viroids cannot replicate in vitro cell culture systems. Considering that there may be no suitable cells for PCVL258 and PCVL264 culture and proliferation in vitro, we constructed molecular cloning of the virus to infect animals for research directly. This study demonstrated that two copies of the complete PCVL258 and PCVL264 genomes in tandem could infect BALB/c mice. The study's findings revealed that PCVLs DNA were detectable to varying degrees in the heart, liver, spleen, lung, and kidney of mice that were inoculated with PCVLs, suggesting that PCVLs have a broad tissue tropism. However, PCV was more commonly detected in the lungs and lymphoid tissues than in other organs. The PCR amplification fragment can be distinguished in a pooled DNA sample using primers binding T7 and T3 and a pair of primers specific for PCVL258 or PCVL264, whether from the carrier or itself. It can also be identified and confirmed by detecting whether the copy number of the virus genome has increased or by nucleic acid in situ hybridization.

The genome of PCV is a single-stranded circular DNA, mainly encoding capsid protein (Cap) and replicase protein (Rep). It has been proposed that PCVs replicate using a rolling circle replication (RCR) model, and the origin of DNA replication (Ori) is represented by a “stem-loop” structure containing a conserved octanucleotide motif sequence (AxTAxTAC). The rep-gene region contains the signature sequences, such as the P-loop, a putative helicase domain with ATPase activity, which can specifically recognize the stem-loop structure for RCR [15, 16]. However, neither porcine circovirus-like viruses nor PCVLs contain rep-gene and stem-loop structures similar to those of PCVs in their genomes [10, 11, 17]. Therefore, it still needs to be made clear how they replicated.

This study used transcriptomics and DIA quantitative proteomics to analyze the biological activities of PCVL258 and PCVL264 systematically. DIA is a new mass spectrometry data acquisition method developed in recent years. It combines the advantages and characteristics of the traditional proteomics “shotgun” method and the mass spectrometry absolute quantitative “gold standard” selective reaction monitoring/multireaction monitoring technology. Compared to the traditional data-dependent acquisition mode, it has the advantages of panoramic scanning, high data utilization, high repeatability, high quantitative accuracy, and data traceability. Therefore, DIA technology can obtain more accurate results [18, 19].

The selection of the liver as the experimental model is based on two key factors. First, the anatomical location of the liver makes it easy to collect, which is conducive to rapid freezing of the collected tissue and minimizing RNA degradation. Second, as the liver is a relatively large organ, it allows for multiple RNA extractions, ensuring the high-quality RNA required for omics analysis. However, it is worth noting that the liver contains different subtypes of cells, such as endothelial cells, Kupffer cells, and hepatocytes [20], making it more meaningful to identify which specific cell types play specific roles.

The results showed that PCVL258 and PCVL264 had multiple biological functions. The DEGs/DEPs after PCVL258 infection in mice were involved in metabolism, cancer, and neurodegenerative disease pathways, and the DEGs/DEPs after PCVL264 infection in mice were involved in metabolic, reproductive, and pancreatic disease pathways. After PCV2 infection, the DEGs were involved in cell cycle regulation and immune response [21, 22] or in the defense response to viruses, cell–cell signaling, and cell proliferation or apoptosis regulation [23].

Due to the inability of viroid RNAs to encode proteins, the invasion, replication, transmission, and evasion of the body's defense barrier of viroids rely on their own RNA sequence/structure motifs for interacting with host proteins. Most viroid RNA molecules exhibit rod-like secondary structures, whereas a few exhibit multibranched secondary structures. They all contain pathogenic determinants [24].

**Figure 8:**
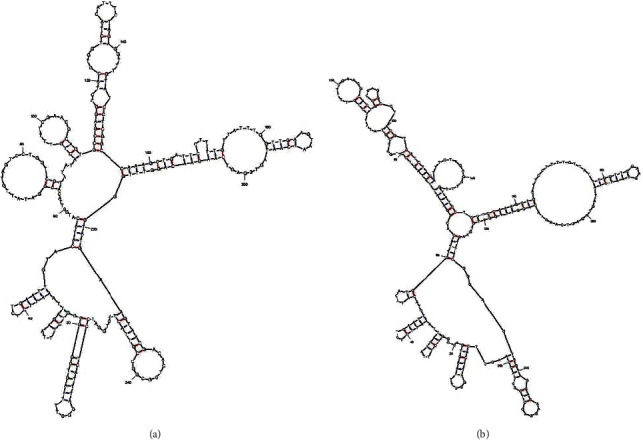
Potential DNA secondary structures of PCVL258 (a) and PCVL264 (b) predicted by Mfold web server, with ∆*G* values of −11.54 and −8.65 kcal/mol, respectively.

Assuming that PCVL258 and PCVL264 are single-stranded DNA genomes similar to those of PCVs and porcine circovirus-like virus P1, the overall secondary structures of PCVL258 and PCVL264 predicted using the Mfold web server (http://www.unafold.org/mfold/applications/dna-folding-form.php) [25] exhibited a Y-shaped structure with multiple small branches, and the three main branches in “Y” were connected by C-G on the central loop (Figure 8). PCVL258 and PCVL264 shared 90.5% nucleotide identity. PCVL258 has two potential ORFs encoding potential functional small peptides (≤17 aa), and the PCVL264 genome also contains two potential ORFs identical to the ORFs of PCVL258. A few signaling pathways, such as metabolic pathways and retinol metabolism, involved in PCVL258 infection are the same as those involved in PCVL264 infection, but most signaling pathways are different. These differences cannot be attributed, or rather cannot be entirely attributed, to the potential peptides encoded by PCVL258 or PCVL264. Therefore, further research is required to determine how different DNA structures, whether overall or local, affect biological functions. Additionally, it remains to be seen whether the genomes of PCVL258 and PCVL264 can be transcribed into RNA to encode peptides.

Total RNA from PK15 cells transfected with the molecular clone of porcine circovirus-like virus P1 was obtained and hybridized to the Affymetrix Porcine GeneChip; the results showed that DEGs were involved in the digestive gland (gastric acid, salivary, and pancreatic) secretion and neurodegenerative diseases (Alzheimer's disease and Huntington's disease). Subsequent studies have confirmed that the P1 virus activates the pancreatic secretion pathway by interacting with its capsid protein and the host M3 receptor [26]. At present, the exact etiology of most neurodegenerative diseases, such as Alzheimer's disease, remains unclear. Since Renvoize first reported that Alzheimer's disease might be related to viruses, an increasing number of studies have shown that viruses such as human cytomegalovirus, herpes simplex type 1, 2, and 6A/B, Epstein–Barr virus, hepatitis C virus, influenza virus, Zika virus, and severe acute respiratory syndrome coronavirus 2 may be involved in the development of Alzheimer's disease [27–29]. Our study also shows that porcine circovirus-like virus P1 and PCVL258, members of the *Circoviridae* family, may be associated with Alzheimer's disease.

Compared with PCV2, the biological functions of these two mini agents were similar to those of porcine circovirus-like virus P1 to some extent. For example, PCVL264 was involved in the pancreatic secretion pathway, whereas PCVL258 was involved in the neurodegenerative disease pathway. These two factors do not encode capsid proteins, and the mechanism involved in this pathway differs from that of the P1 virus. Although these two agents cannot encode proteins, there are small ORFs in their genomes. It remains unclear whether they encode peptides. However, even if a peptide is encoded, the above biological functions cannot be completely attributed to the peptides encoded by the agents because the same ORFs exist. Viroid pathogenicity primarily results from the structural complexity of viroid-derived small RNAs in RNA silencing pathways [30]. It is unclear whether PCVLs have pathogenic mechanisms similar to those of viroids.

In conclusion, to the best of our knowledge, this study represents the first attempt to analyze the biological activities of PCVL258 and PCVL264 using transcriptomics combined with proteomics. Integrated proteomics and transcriptomics analysis revealed that PCVL258 and PCVL264 infection modulate various host BPs, including metabolic, reproductive, and human disease pathways. This knowledge may help characterize these pathogen-associated DNA molecules.

## Figures and Tables

**Table 1:** Primer sequences for qRT-PCR validation.

Gene name	Primer sequence (5ʹ→3ʹ)	Amplicon size	Accession number
Try4	F:tacaagtcccgcatccaagt R:attgagggtcacaggggaag	168	BC061135

H4c17	F:tcttcgtcatgtctggtcgt R:cgtagatgaggccggagatg	165	NM001195421

Prss2	F:atgctggctaccacttctgt R:cgtagatgaggccggagatg	236	NM009430

H2bc3	R:ggtcgagcgcttgttgtaat F:aaagaggagtgtggaggagc	203	NM175664

Cy2b10	F:aaagaggagtgtggaggagc R:tgtactcaaagcgctctcca	158	BC060973

Gsta1	F:aagagaagccaagactgcct R:ttcttcacattggggaggct	240	NM008181

Hba-a2	F:tgaagccctggaaaggatgt R:tgaagttgacgggatccaca	218	NM001083955

Cyp17a1	F:acctaatgccaagttcccca R:ctctggccagctgatagtga	185	NM_007809

**Table 2:** Distribution of PCVL258 and PCVL264 in control and PCVL-inoculated mice.

Group	Inoculum	DPI	Heart	Liver	Spleen	Lung	Kidney
C	pSK	3	0/3	0/3	0/3	0/3	0/3
		7	0/3	0/3	0/3	0/3	0/3
		14	0/3	0/3	0/3	0/3	0/3
		21	0/3	0/3	0/3	0/3	0/3
		28	0/3	0/3	0/3	0/3	0/3

T	PCVL264 DNA	3	3/3	3/3	3/3	3/3	3/3
		7	3/3	3/3	3/3	3/3	3/3
		14	2/3	2/3	2/3	1/3	1/3
		21	3/3	2/3	2/3	3/3	2/3
		28	1/3	1/3	1/3	1/3	1/3

TII	PCVL258 DNA	3	3/3	3/3	3/3	3/3	3/3
		7	3/3	3/3	3/3	3/3	3/3
		14	2/3	1/3	1/3	1/3	1/3
		21	1/3	1/3	2/3	1/3	2/3
		28	1/3	1/3	1/3	1/3	1/3

*Note*. Three mice from each group were necropsied at 3, 7, 14, 21, and 28 DPI. Number positive/number tested.

**Table 3:** qRT-PCR validation of the expression of selected genes from RNA-seq between control and PCVL258-infected BALB/c mice.

Gene	RNA-seq FC	qRT-PCR FC
Try4	8.11	6.90
H4c17	69.55	72.28
Prss2	9.25	8.33
H2bc3	153.27	170.00
Cy2b10	0.15	0.19
Gsta1	0.05	0.06
Hba-a2	0.27	0.30
Cyp17a1	0.16	0.14

## Data Availability

The transcriptome sequencing data are available at the NCBI Sequence Read Archive (SRA) database (no. PRJNA849731). The mass spectrometry proteomics data have been deposited to the ProteomeXchange Consortium (http://proteomecentral.proteomexchange.org) via the iProX partner repository with the dataset identifier PXD039230.
